# Homocysteinemia as a cause for amaurosis fugax in a patient without an apparent embolic source

**Published:** 2019

**Authors:** Sibel Inan, Guliz Yavas, Umit Ubeyt Inan

**Affiliations:** * Department of Ophthalmology, Health Sciences University Medical School, Afyon, Turkey; ** Department of Ophthalmology, Hacettepe University Medical School, Ankara, Turkey; *** Department of Ophthalmology, ParkHayat Hospital, Afyon, Turkey

**Keywords:** Transient Visual Loss, homocysteinemia, recurrent attacks

## Abstract

**Purpose:** To report a case with monocular transient vision loss (TVL) associated with Hyperhomocysteinemia.

**Methods:** We present a case with persistent TVL attacks and high level of homocysteine.

**Results:** A 32-year-old male had a history of episodes of recurrent monocular TVL. Extensive ophthalmic, systemic and laboratory studies were unremarkable with the exception of high plasma homocysteine level. He never experienced TVL during the 36-month follow-up after starting folate, B12 and B6 except for one episode in which he had discontinued the treatment for three months.

**Conclusion:** This case may suggest hyperhomocysteinemia as one of the underlying causes of recurrent attacks of TVL without any known source of emboli.

## Introduction

Homocysteine is a sulfur amino acid produced by the metabolism of methionine, which is one of the essential amino acids obtained from dietary proteins. Homocysteine is converted to methionine or cysteine via remethylation or transsulfuration, respectively. Genetic mutations in the enzymes of this pathway can contribute to an increase in plasma homocysteine levels [**1**-**3**]. 

Homocysteinemia is an independent risk factor for cardiovascular disease. Hyperhomocysteinemia has been suggested as an independent risk factor for atherosclerotic cardio-vascular disease [**1**,**2**]. It can increase the risk for arterial and venous thrombosis and may be an important risk factor in premature deaths due to vascular pathologies [**2**]. So, homocysteinemia may be related to premature atherosclerosis and thromboembolism of crucial vascular structures such as coronary arteries, extracranial and intracranial cerebral arteries and veins, and the peripheral arteries and veins. Hyperhomocysteinemia has statistically found to be more prevalent in patients with retinal vein occlusions like central retinal artery occlusion and central and branch retinal vein occlusion [**3**-**7**]. 

Plausible mechanisms to mediate a deleterious effect of high homocysteine level include vascular endothelial dysfunction, promotion of oxidation of low-density lipoprotein cholesterol, vascular smooth cell proliferation, and coagulation abnormalities [**1**,**2**]. Abnormalities of platelet function, thrombin generation or fibrinolysis may be related to thrombosis associated with hyperhomocysteinemia [**1**-**3**]. 

Amaurosis fugax is defined as transient monocular vision loss lasting seconds to minutes and is thought to be caused by emboli of fibrin-platelet aggregates derived from atheromatous lesions of the carotid artery or from the cardiac valves [8-**10**].

Although homocysteinemia has been suggested as a cause of retinal vascular occlusion and stroke, to the best of our knowledge, a direct association of amaurosis fugax and homocysteinemia without any occlusive disease has not been documented. We present a case with persistent amaurosis fugax attacks caused by high level of homocysteine.

## Case Report

The reported case was a doctor employed in an academic institution. The male patient applied for his newly experienced symptoms of monocular transient vision loss. He was thirty-two years old. He had a previous history of recurrent amaurosis fugax attacks lasting 2-3 minutes one at every 2 or 3 months for 7 years. His best-corrected visual acuity was 20/ 20 in each eye. Slit lamp examination showed normal anterior segment findings. Dilated fundus examination was normal. One week later, he complained of a new symptom of complete amaurosis that started half an hour before, lasted one minute, and resolved gradually within 5 minutes in his left eye. He underwent fundus fluoresceine angiography, and there was no abnormality, except for relatively delayed choroidal filling. In color fundus photography of the left eye, a localized narrowing in supero-temporal branch of the retinal artery suggesting vasospasm was observed (**Fig. 1**). Color Doppler examination of carotid and vertebral arteries was normal. Color Doppler imaging was also performed in the central retinal artery, ophthalmic artery, short posterior ciliary arteries and no blood flow abnormality was observed. Specifically, there was no history of hypertension, diabetes, hyperlipidemia, or clotting disorder. He had been smoking 10 cigarettes daily for the past 8 years. There was no personal or family history of migraine and cardiovascular disorder. Neuro-ophthalmologic examination was completely normal. His cardiovascular assessment including systolic and diastolic blood pressure, physical examination, electrocardiogram, and transesophageal echocardiography exposed no noteworthy findings. 

**Fig. 1:**
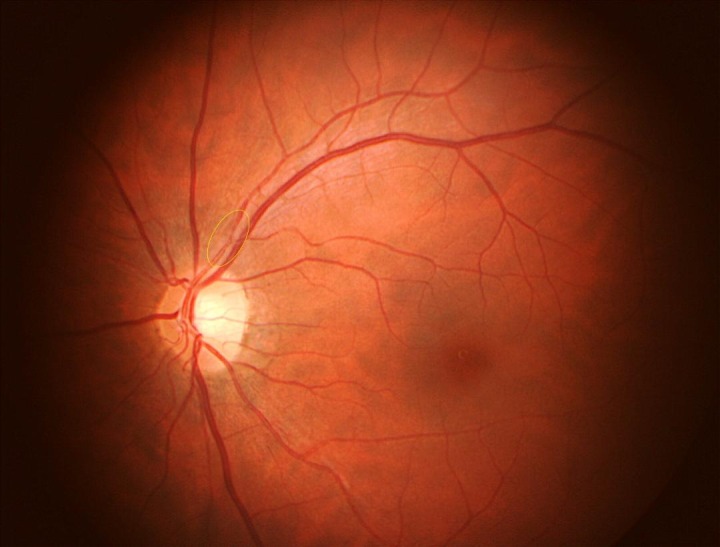
Color fundus photography of the left eye. A localized narrowing is seen in the supero-temporal branch of the retinal artery

In whole laboratory investigation, hemogram, fibrinogen, prothrombin time, partial thromboplastin time, Anti-cardiolipin IgM and IgG, anti-thrombin III activity, protein C activity, Active protein-C resistance, free protein S, C3, C4, RF, IgG, IgM, Ceruloplasmin, Alpha1-antitrypsin, haptoglobin, anti-streptolysin-O, C-reactive protein, fasting blood glucose, hepatic and renal function tests, electrolytes were found to be within normal limits. After first laboratory examination serum homocysteine, vitamin B12 and folate levels were studied. Serum fasting homocysteine level was above the upper limits (18.95 μmol/ L; laboratory range, 5-15 μmol/ L). Serum vitamin B12 level was 185 pg/ mL (laboratory range; 211-911 pg/ mL) and folate level was 4.9 ng/ mL (laboratory range; 3.1-20 ng/ mL). The etiology of the amaurosis fugax was considered to be due to homocysteinemia and treatment with vitamin B12, B6 and folate was commenced. 

Four months later, he complained of transient gaze palsy, diplopia and ptosis that continued for several hours. On ophthalmic examination, ptosis was mild, and gaze palsy and diplopia had resolved. Pupils were equal and normally reactive. Extraocular muscle movements were intact, and the patient had normal ocular alignment in oculomotor examination. The eyes were ortophoric in cover test. Convergence was also normal. No other ocular signs were observed. Magnetic resonance imaging of hypophysis showed a nonspecific and lobulated lesion in the right side of dorsum sella lying into sphenoid sinus and destructing the bone contour in T1W section (**Fig. 2**, bottom left). No contrast enhancement was observed after the repetition of MRI with gadolinium. Multiple small infarct areas were also observed in the head of the left caudate nucleus and right centrum semiovale (**Fig. 2**, top left and right). In cerebral MR angiography, P1 branch of the left posterior cerebral artery was not seen (**Fig. 2**, bottom right). There was no other abnormality in MR angiography. The lesion was stable in control MRI examinations. The abnormality in the left posterior cerebral artery was considered as a variety. 

**Fig. 1:**
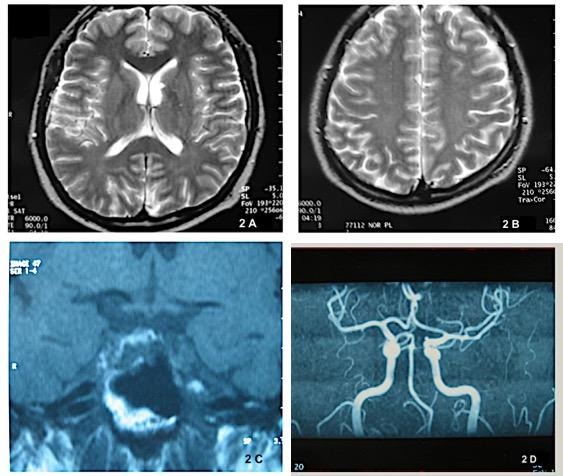
**Top left (A)**: T2 W MRI of the brain shows a hyperintensity consisted with lacunar infarct in the head of caudate nucleus and adjacent to the ventricle. **Top right (B)**: T2 W MRI of brain shows three hyperintense areas consistent with lacunar infarct of white matter in the level of centrum semiovale. **Bottom left (C)**: T1 W coronal plane MRI of hypophysis demonstrates an isointense mass in the right half side of the hypophysis lying into sphenoid sinus, displaying osseous destruction and does not show an enhancement of contrast agent. **Bottom right (C)**: 3D-TOF MRI Angiography demonstrates absence of P1 branch of the left posterior cerebral artery

The patient did not develop attacks of transient visual loss until month 20. He also did not develop diplopia or ptosis attack during this period. At month 24, serum fasting homocysteine level was in normal range (5.25 μmol/ L) with supplementation of folate, vitamin B6, and vitamin B12. At month 30, he experienced visual disturbance while talking with his colleagues in their department. At that moment, his colleagues noticed a horizontal nystagmus lasting about 2 minutes. He applied to us 20 minutes after this incident. His ophthalmic and neurological examination was completely normal. He confessed that he did not go on vitamin treatment for three months, and started his treatment again. 

## Discussion

We described a case experiencing recurrent attacks of amaurosis fugax at approximately every 2 or 3 months for the past 7 years. There was no notable finding in cardiovascular or extensive laboratory investigations to explain the underlying etiology except for mildly elevated serum homocysteine level. The patient responded to treatment with vitamin supplementation and he did not experience any attacks of TVL under the treatment during the 36 months follow-up. The patient presented with ptosis and diplopia 4 months following the initiation of the treatment. A brain tumor in dorsum sella was established with MRI examination. The mass was bone related and considered as benign therefore, surgical intervention was not recommended. The source of the ptosis and diplopia was unclear, but the patient did not develop ptosis or diplopia during follow-up. Consecutive presentation of the brain tumor was probably coincidental and we did not consider any association with TVL attacks.

A sudden temporary cessation of the blood flow to one eye can cause amaurosis fugax, as a monocular TVL. The vision loss is abrupt and usually lasts seconds or minutes. Blindness is complete, although sometimes limited to a sector of the visual field. The vision is usually described as “dark” or “dim”. The return of vision can be sectorial or altitudinal and is occasionally described as a “curtain rising”. Generally, the vision returns to normal immediately after an attack. The frequency of these attacks varies from 1 or 2 attacks per month to 10 or 20 per day [**8**-**10**]. The causes of monocular TVL in young individuals include vasculitis, embolization from cardiac disease, particularly mitral valve prolapse, use of oral contraceptives, and thrombophilia conditions such as protein C and S deficiency, anti-thrombin III deficiency, factor V Leiden mutation, prothrombin gene mutation, and methylenetetrahydrofolate reductase mutation [**11**-**14**]. Aortic or cardiac vascular abnormality was not found in our patient’s carotid. Investigation revealed no evidence of an embolic or atheromatous etiology. An association with increased antiphospholipid antibodies, hyperviscosity and hypercoagulable states, polycythemia vera, systemic lupus erythematosus, and arterio-venous malformations could not be demonstrated. Homocysteine level was 18.95 µmol/ L in our case. Homocysteine level between 15-30 µmol/ L has been referred to mild hyperhomocysteinemia [**7**]. Homocysteinemia was considered responsible for the amaurosis fugax, because of normal laboratory investigation except for increased serum homocysteine levels. The role of hypercoagulable states in the etiology of TVL is unknown. Thrombophilia testing after TVL should be done in patients with recurrent TVL, a personal or family history of thrombotic events [**9**]. Due to the reversible nature of hyperhomocysteinemia, testing for its serum levels may be beneficial. Reduction of serum homocysteine levels to normal values after a median follow-up of 21 months after treatment with folic acid, vitamin B6, and vitamin B12 has been demonstrated in patients with TVL [**13**]. 

Amaurosis fugax has also been associated with migraine. Although migraine has been suggested as a possible cause for transient visual loss in young patients, our case did not have a history of migraine. Vasospasm may be another cause of TVL [**15**]. Vasospasm has been reported as a manifestation of homocysteinemia. Homocysteinemia can cause an abnormality in magnesium metabolism of cerebral vascular smooth muscle cells, thus priming these cells for atherogenesis, cerebral vasospasm and stroke [**14**,**15**]. Either thrombosis or vasospasm, which can both be induced by homocysteinemia, may be a causative factor for TVL in our case. Multiple small infarct areas in T2W section of brain MRI showed numerous transient ischemic attacks probably due to thromboembolic event or vasospasm. An appearance of vasospasm in the superior branch retinal artery of our patient may also be a subtle sign suggesting this consideration.

The compromising event in our case was the diagnosis of a brain tumor after sudden onset and transient presentation of mild ptosis and diplopia. The localization of the tumor did not give rise to an opinion that the tumor was the cause of the TVLs. Small infarct areas in brain MRI indicate multiple transient ischemic attacks, so confirm previously experienced recurrent attacks of TVL. Transient ischemia is possible to produce transient diplopia or ptosis. Transient horizontal nystagmus may also be caused by the transient ischemic attack of the brainstem [**8**]. Thus, it is possible that all the symptoms presented by our case are due to homocysteinemia related vascular etiology. 

Our case suggests that elevated serum homocysteine may be a causal factor and may be in place among the etiologies of TVL, especially in young patients. Hyperhomocysteinemia can easily be treated with dietary supplementation of folate, vitamins B12 and B6. Studies are needed to reveal an association of homocysteinemia with TVL in different age groups. 

**Patient Consent**

Written patient consent to publish the report was obtained. 

**Funding**

No funding or grant support. 

**Conflict of Interest**

There is no conflict interest.

**Authorship**

All authors attest that they met the current ICMJE criteria for authorship. 

**Acknowledgements**

None. 
